# Neoadjuvant Chemotherapy Followed by Laparoscopic and Endoscopic Cooperative Surgery for Locally Advanced Duodenal Gastrointestinal Stromal Tumor: A Case Report

**DOI:** 10.70352/scrj.cr.25-0486

**Published:** 2025-10-10

**Authors:** Kazuya Takabatake, Takeshi Kubota, Naoto Iwai, Osamu Dohi, Hiroyuki Inoue, Kento Kurashima, Keiji Nishibeppu, Jun Kiuchi, Hidemasa Kubo, Taisuke Imamura, Kenji Nanishi, Hiroki Shimizu, Tomohiro Arita, Toshiyuki Kosuga, Yusuke Yamamoto, Hirotaka Konishi, Ryo Morimura, Hitoshi Fujiwara, Atsushi Shiozaki

**Affiliations:** 1 Division of Digestive Surgery, Department of Surgery, Kyoto Prefectural University of Medicine, Kyoto, Kyoto, Japan; 2 Molecular Gastroenterology and Hepatology, Graduate School of Medical Science, Kyoto Prefectural University of Medicine, Kyoto, Kyoto, Japan

**Keywords:** duodenal gastrointestinal stromal tumor, laparoscopic and endoscopic cooperative surgery, neoadjuvant chemotherapy

## Abstract

**INTRODUCTION:**

Duodenal gastrointestinal stromal tumors (D-GISTs) are rare neoplasms that pose surgical challenges, particularly when located near critical structures such as the ampulla of Vater or the pancreatic head. Although pancreaticoduodenectomy (PD) is often required in such cases, it is associated with significant morbidity. Neoadjuvant chemotherapy (NAC) with imatinib has emerged as a strategy to downsize tumors, thereby enabling organ-preserving resections. Laparoscopic and endoscopic cooperative surgery (LECS), initially developed for gastric submucosal tumors, has been adapted for duodenal lesions (D-LECS) in selected cases. Here, we present a case in which neoadjuvant imatinib therapy enabled local resection using the D-LECS approach.

**CASE PRESENTATION:**

A 67-year-old man was diagnosed with a 55-mm D-GIST involving the duodenal bulb and descending portion, located near the ampulla of Vater and pancreatic head. NAC with imatinib (400 mg/day) was administered for 2 months, resulting in significant tumor shrinkage to 27 mm, without invasion of adjacent structures. D-LECS was performed using an endoscopic submucosal incision followed by laparoscopic full-thickness resection. After laparoscopic suturing, minor leakage at the end of the suture line was reinforced using the reopenable-clip over line method. The procedure was completed safely with negative margins and no complications. The patient was discharged on POD 14, and remained recurrence-free at 12 months.

**CONCLUSIONS:**

Neoadjuvant imatinib effectively downsized a locally advanced D-GIST, enabling safe, minimally invasive resection via D-LECS. This case highlights the potential of NAC and D-LECS as a less invasive alternative to PD in anatomically challenging D-GISTs.

## Abbreviations

D-GIST
: duodenal gastrointestinal stromal tumor
EGD
: esophagogastroduodenoscopy
LECS
: laparoscopic and endoscopic cooperative surgery
NAC
: neoadjuvant chemotherapy
PD
: pancreaticoduodenectomy
ROLM
: reopenable-clip over line method

## INTRODUCTION

D-GISTs are relatively rare, accounting for approximately 3%–5% of all GISTs.^1)^ The standard treatment for GIST is complete surgical resection. As systematic lymph node dissection has not been shown to improve outcomes, organ-preserving local resection is generally considered the preferred approach, including for D-GISTs.^2)^ However, depending on the tumor’s size and anatomical location, especially when it is adjacent to the ampulla of Vater or the pancreatic head, achieving complete resection may require more extensive procedures such as PD.^3)^

Recently, NAC using imatinib has been reported to be beneficial in cases involving large tumors or those at high risk for incomplete resection.^4)^ NAC may facilitate tumor downsizing, thereby increasing the likelihood of performing local resection and preserving organ function. In D-GISTs, in particular, several reports have suggested the utility of NAC as a strategy to enable less invasive, organ-preserving surgery.^5,6)^

In addition, LECS has been established as a minimally invasive technique for the resection of GISTs.^7)^ While originally developed for gastric lesions, its application has been extended to duodenal lesions in selected cases.^8)^ Here, we report a case of D-GIST in which preoperative imatinib therapy allowed for local resection using duodenal LECS (D-LECS), avoiding the need for PD and laparotomy.

## CASE PRESENTATION

**Fig. 1:**
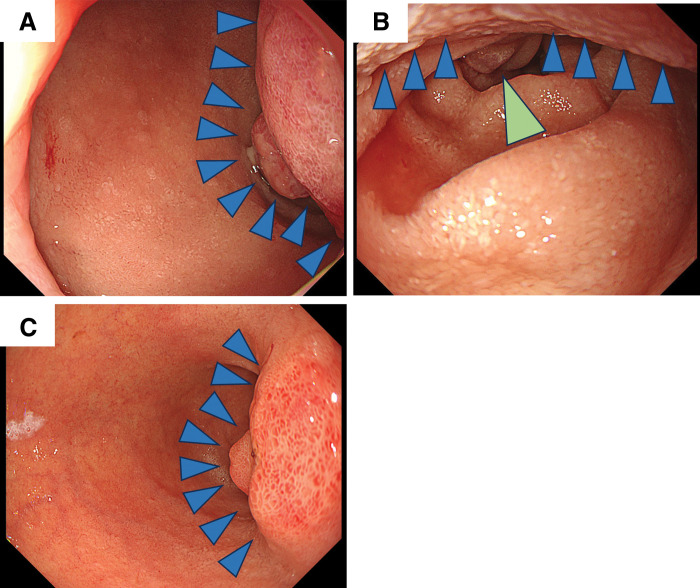
Endoscopic findings before and after neoadjuvant chemotherapy. (**A**) The tumor was located on the posterior wall of the duodenal bulb. (**B**) The tumor was adjacent to the ampulla of Vater. (**C**) After imatinib therapy, the size of the tumor decreased. Blue arrowheads indicate the tumor, and the light green arrowhead indicates the ampulla of Vater.

**Fig. 2:**
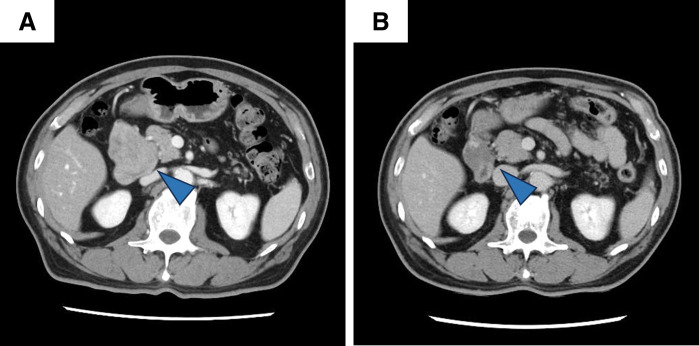
Enhanced CT findings before and after neoadjuvant chemotherapy. (**A**) A 55-mm tumor was located in the duodenal bulb. (**B**) After imatinib therapy, the tumor size decreased to 27 mm. No evidence of pancreatic invasion was observed. Blue arrowheads indicate the tumor.

A 67-year-old male was referred to our department for further management of D-GIST. The patient presented to a previous hospital with dyspnea and anemia. EGD revealed a submucosal tumor with *delle* extending from the posterior wall of the duodenal bulb to the descending portion near the ampulla of Vater (**Fig. 1A**, **1B**). Histopathological examination of biopsy specimens confirmed the diagnosis of c-kit–positive GIST. Abdominal contrast-enhanced CT demonstrated a 55-mm enhancing mass in the descending duodenum (**Fig. 2A**). ^18^F-fluorodeoxyglucose PET showed abnormal uptake localized to the tumor, with no signs of distant metastasis. Given the relatively large tumor size and its proximity to the ampulla of Vater and the pancreatic head, open PD was initially considered. However, because of the possibility of local resection in case of shrinkage of the tumor, imatinib mesylate (400 mg/day) was initiated as neoadjuvant therapy settings for 2 months. The treatment was well tolerated, except for grade 2 neutropenia, dysgeusia, and oral mucositis. Subsequent imaging revealed a marked reduction in tumor size to 27 mm, without evidence of invasion into adjacent organs (**Fig. 2B**). EGD confirmed that the tumor was localized to the posterior wall of the duodenal bulb (**Fig. 1C**). Local resection was deemed feasible, and D-LECS was performed.

**Fig. 3:**
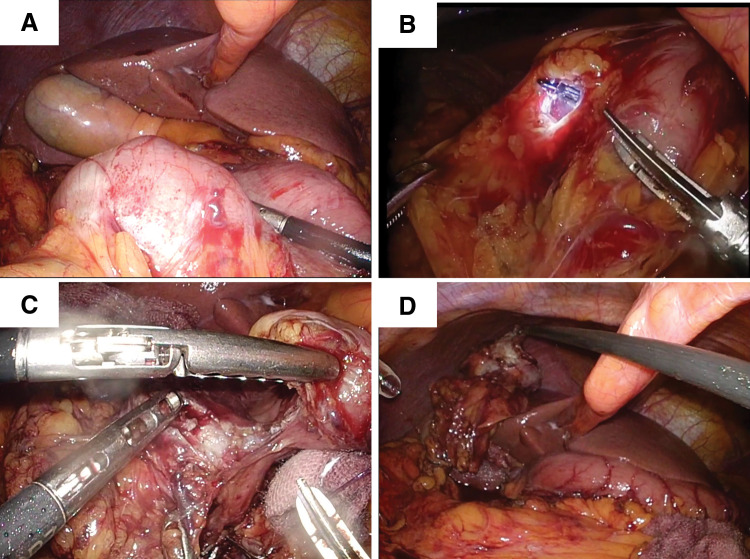
Operative findings. (**A**) The tumor was located in the duodenal bulb. (**B**) After circumferential endoscopic dissection of the mucosal and submucosal layers around the tumor, approximately half of the duodenal circumference was dissected in a full-thickness manner through a combination of endoscopic and laparoscopic approaches. (**C**) The tumor was carefully separated from the pancreas and excised in a full-thickness manner along the endoscopic markings. (**D**) Complete dissection of the tumor was achieved.

Under general anesthesia, the patient was placed in the supine position with the lower limbs abducted. The abdominal cavity was accessed via a 15-mm umbilical incision using Hasson’s technique, and pneumoperitoneum was established with the placement of a 12-mm blunt port. Both laparoscopic and endoscopic examinations identified a tumor on the posterior wall of the duodenal bulb (**Fig. 3A**). Additional ports were placed in the bilateral subcostal and lateral abdominal regions. The greater omentum was incised toward the hepatic flexure of the colon, exposing the 2nd portion of the duodenum. Circumferential incision of the mucosal and submucosal layers around the tumor was then performed using endoscopic submucosal dissection techniques. Upon reaching the serosal layer, full-thickness dissection of approximately half the circumference of the duodenum was completed through a combination of endoscopic and laparoscopic approaches (**Fig. 3B**).

**Fig. 4:**
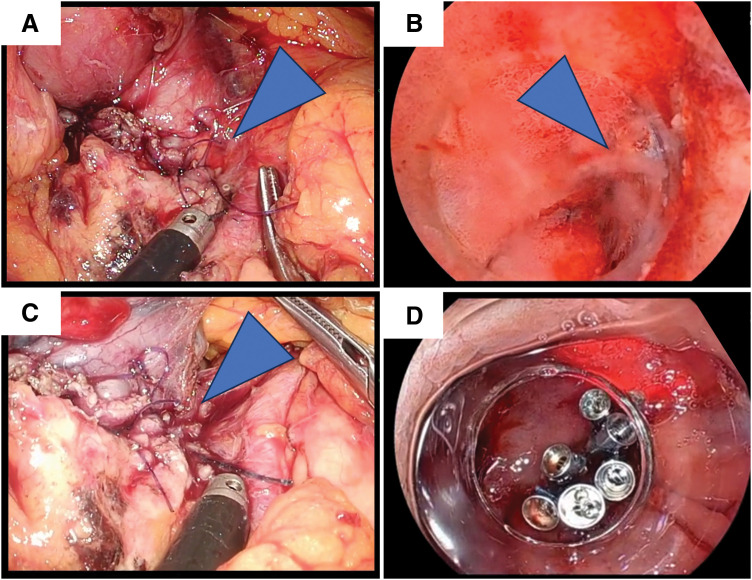
Suturing using combined laparoscopic and endoscopic techniques. (**A**, **B**) After a single-layer full-thickness suture, leakage was observed at the end of the suture line near the pancreas. (**C**, **D**) The leakage was repaired by endoscopic suturing using the “reopenable-clip over line method.” Blue arrowheads indicate the point of leakage.

**Fig. 5:**
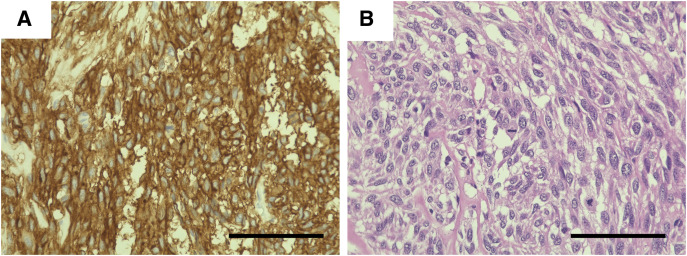
Histopathological findings. (**A**) Immunohistochemical staining shows c-kit positivity. (**B**) The edematous and hyalinized areas, which accounted for approximately 30% of the entire tumor, suggested a potential histological response to neoadjuvant imatinib therapy. Scale bars indicate 100 μm.

Subsequently, the right gastroepiploic vessels were divided laparoscopically to gain access to the tumor. The tumor was carefully dissected away from the pancreas and excised in a full-thickness fashion along the endoscopic markings (**Fig. 3C**, **3D**). To prevent intraperitoneal dissemination, the specimen was immediately enclosed in a retrieval bag after resection and extracted from the abdominal cavity. The duodenal defect was closed in the short-axis direction using a single-layer, full-thickness suture. Endoscopic examination confirmed no narrowing of the duodenal lumen; however, minor leakage was observed at the end of the suture line (**Fig. 4A**, **4B**). Given the close proximity to the pancreas and the gastroduodenal artery, laparoscopic suturing was deemed challenging. Therefore, the “ROLM,” an endoscopic suturing technique, was employed to reinforce closure from the luminal side (**Fig. 4C**, **4D**). After confirming the absence of leakage and bleeding, the suture site was covered with the omentum. Finally, drains were placed anterior and posterior to the duodenum. The total operative time was 240 min, consisting of 150 min for the laparoscopic procedures and 90 min for the endoscopic procedures, including ROLM. Intraoperative blood loss was 100 g. Histopathological examination revealed a c-kit–positive GIST with 20 mitoses per 50 high-power fields and negative surgical margins (**Fig. 5A**). Accordingly, the tumor was classified as a high-risk GIST. Edematous and hyalinized areas—indicative of a potential histological response to chemotherapy—comprised approximately 30% of the tumor (**Fig. 5B**). There were no postoperative complications, including stenosis, and the patient was discharged on POD 14. At the 12-month follow-up, there was no evidence of tumor recurrence.

## DISCUSSION

This case highlights the effectiveness of NAC with imatinib and a laparoscopic–endoscopic cooperative approach in achieving minimally invasive, organ-preserving resection for D-GIST. The patient initially presented with a 55-mm tumor near the ampulla of Vater and the pancreatic head, for which PD was considered. After 2 months of NAC, the tumor decreased by 51% and became clearly separable from these critical structures, permitting anticipated negative margins. This downsizing allowed safe resection using D-LECS and avoided the morbidity associated with PD, although PD was prepared as a backup and would have been undertaken if pancreatic invasion, ampullary involvement, or failure of secure closure had been encountered.

Imatinib-based NAC has been shown to improve resectability and facilitate organ preservation in gastric GISTs. Kurokawa et al. demonstrated that in large gastric GISTs, 6–9 months of preoperative imatinib achieved R0 resection rates of up to 91% and organ preservation rates of 88%.^4)^ While data for NAC in D-GISTs are more limited, several retrospective reports have demonstrated its ability to downsize tumors and avoid extended resections such as PD.^5,6)^ Lv et al. reported a series of 10 patients with locally advanced D-GISTs in which 2–18 months of NAC with imatinib led to significant tumor shrinkage and enabled organ-preserving surgery in 90% of cases, with tumor response evaluated every 2 months after NAC initiation.^6)^ While the mean NAC duration was approximately 7 months in their series, our patient discontinued the treatment at 2 months due to grade 2 toxicities. Nevertheless, the tumor shrank by 51%, allowing organ-preserving resection. Importantly, they also documented 1 case that achieved successful downstaging after only 2 months of imatinib. These findings suggest that even short-course NAC may provide an optimal balance between cytoreduction and treatment-related toxicity in anatomically constrained D-GISTs.

LECS was originally introduced for gastric submucosal tumors and has since been applied to duodenal lesions. Ichikawa et al. reported a 100% R0 rate, no Clavien–Dindo grade ≥III complications, and no postoperative strictures in non-ampullary duodenal epithelial tumors treated with D-LECS.^8)^ Although evidence for D-LECS in submucosal tumors such as Brunner’s gland hamartomas and GISTs remains limited, published cases have demonstrated favorable outcomes.^9–11)^ Consistent with these reports, the present operation was completed in 240 min with 100 mL blood loss, and the patient was discharged on POD 14 without any complications. Compared with open and extended resection, D-LECS offers superior preservation of duodenal continuity and lower invasiveness when anatomically feasible.

A unique challenge of D-LECS is the secure closure of the duodenal wall after resection, especially when the tumor is near major vascular structures or the pancreas. In our case, closure with laparoscopic suturing was deemed difficult due to the tumor’s proximity to the pancreatic head and the gastroduodenal artery. We therefore employed the ROLM, a technique in which a pre-looped nylon suture is cinched by reopenable clips to approximate mucosal defects from the luminal side, and which has achieved a 100% complete-closure rate for gastric defects up to 85 mm.^12)^ In this case, ROLM enabled safe intraluminal closure of the defect adjacent to critical structures. To our knowledge, this is the 1st report of ROLM applied during D-LECS. Successful D-LECS thus depends on both advanced laparoscopic skills and sophisticated endoscopic techniques, underscoring the need for close collaboration with experienced surgeons and endoscopists.

## CONCLUSIONS

This case demonstrates that neoadjuvant imatinib can downstage locally advanced, papilla-adjacent D-GIST, expanding the indications for minimally invasive, organ-preserving resection such as D-LECS. Because procedures in this region are technically demanding and anatomically constrained, their success depends on a multidisciplinary team of experienced surgeons and endoscopists. Continued accumulation of collaborative experience will be essential to optimize patient selection and operative strategies for these challenging tumors.
